# Positive selection on a bacterial oncoprotein associated with gastric cancer

**DOI:** 10.1186/1757-4749-3-18

**Published:** 2011-11-11

**Authors:** Gisela Delgado-Rosado, Maria Gloria Dominguez-Bello, Steven E Massey

**Affiliations:** 1 Biology Department, University of Puerto Rico - Rio Piedras, PO Box 23360, San Juan, Puerto Rico, USA 00931

**Keywords:** gastric cancer, oncogene, positive selection, *Helicobacter pylori*, cagA

## Abstract

**Background:**

*Helicobacter pylori *is a vertically inherited gut commensal that is carcinogenic if it possesses the *cag* pathogenicity island (*cag *PaI); infection with *H.pylori *is the major risk factor for gastric cancer, the second leading cause of death from cancer worldwide (WHO). The *cag *PaI locus encodes the *cagA *gene, whose protein product is injected into stomach epithelial cells via a Type IV secretion system, also encoded by the *cag *PaI. Once there, the cagA protein binds to various cellular proteins, resulting in dysregulation of cell division and carcinogenesis. For this reason, cagA may be described as an oncoprotein. A clear understanding of the mechanism of action of cagA and its benefit to the bacteria is lacking.

**Results**

Here, we reveal that the *cagA *gene displays strong signatures of positive selection in bacteria isolated from amerindian populations, using the Ka/Ks ratio. Weaker signatures are also detected in the gene from bacteria isolated from asian populations, using the Ka/Ks ratio and the more sensitive branches-sites model of the PAML package. When the *cagA *gene isolated from amerindian populations was examined in more detail it was found that the region under positive selection contains the EPIYA domains, which are known to modulate the carcinogenicity of the gene. This means that the carcinogenicity modulating region of the gene is undergoing adaptation. The results are discussed in relation to the high incidences of stomach cancer in some latin american and asian populations.

**Conclusion**

Positive selection on cagA indicates antagonistic coevolution between host and bacteria, which appears paradoxical given that cagA is detrimental to the human host upon which the bacteria depends. This suggests several non-exclusive possibilities; that gastric cancer has not been a major selective pressure on human populations, that cagA has an undetermined benefit to the human host, or that horizontal transmission of *H.pylori *between hosts has been more important in the evolution of *H.pylori *than previously recognized, reducing the selective pressure to lower the pathogenicity of the bacteria. The different patterns of adaptation of the gene in different human populations indicates that there are population specific differences in the human gut environment - due either to differences in host genetics or diet and other lifestyle features.

## Introduction

*Helicobacter pylori *is a Gram negative bacterium that lives in the human stomach as part of the normal gastric microbiome [1], and is generally present in the majority of the adult population [2]. The bacterium has co-evolved with human populations [3] and is well adapted and largely specific to the human host. The ancestor of *H.pylori *was intestinal and during its evolution migrated to the stomach, facilitated by the evolution of a urease that combats the stomach's acid conditions [4,5]. *H.pylori *strains may possess a *cag *pathogenicity island (*cag *PaI) that contains a *cagA *gene encoding a 128 kDa protein [6,7]. The *cag *PaI seems to have entered the *H.pylori *genome by lateral gene transfer, after *H.pylori *differentiated from parental species [2,8]. Many of the genes of the *cag *PAI are involved in translocation of the cagA protein into epithelial cells lining the stomach. However, the function of the cagA protein itself is unknown. Infection with *cagA+ H.pylori *is strongly associated with gastric carcinoma [9-11]; gastric carcinoma is the second leading cause of death from cancer worldwide [12]. In addition, *cagA*^+ ^*H.pylori *is associated with chronic gastritis and peptic ulcers [13].

The mechanism of pathogenicity of *cagA+ H.pylori *is as follows. The bacteria attaches to the stomach wall and the cagA protein is injected into an epithelial cell by a bacterial Type IV secretion system, also encoded by the *cag *PaI locus [14]. Once inside the cell, cagA is phosphorylated on tyrosine residues located within EPIYA domains by members of the src kinases such as c-src, Fyn, Yes [15], Lyn [16] and c-Abl [17]. The cagA protein is membrane associated and interacts with numerous additional cellular proteins, including the oncoprotein Src homology 2 domain containing tyrosine phosphatase (SHP-2 [18]), microtubule affinity-regulating kinase (MARK2 [19]), growth factor receptor-bound protein 2 (Grb-2 [20]), hepatocyte growth factor receptor (c-Met [21]), C-terminal Src kinase (Csk [22]) and p38 (Crk [23]). Tyrosine phosphorylated cagA recruits and activates SHP-2, apparently mimicking the action of Gab1 [24]. Consistent with the mimicry hypothesis, *cagA *is able to rescue Gab1 deficient *Drosophila *mutants [25], which is interesting given that *cagA *has no sequence similarity with Gab1, indeed it has no known homologs. The interaction with SHP-2 causes inhibition of its tumor suppressing activity [18]. Epithelial cells that have been dysregulated adopt the elongated hummingbird phenotype [26]. In addition, cagA activates the transcription factor NF-kB leading to the induction of interleukin 8 (IL-8) and subsequent inflammation [27]. The activation of NF-kB occurs via SHP-2.

Variation in the EPIYA domains of cagA results in variation in the virulences of different *cagA+ H.pylori *strains [28]. The EPIYA motifs are located in the C-terminal half of the cagA protein and are of types A-D. The EPIYA motifs are the major sites of tyrosine phosphorylation within the cagA protein. The eastern EPIYA-D motif, found in asian populations, is associated with stronger binding to SHP-2, while the western EPIYA-C motif is not. The presence of the EPIYA-D motif in asian *cagA *sequences may be responsible for the high rates of *H.pylori *associated disease in asian populations [28].

The study reported here investigates the evolutionary dynamics of the *cagA *gene from different human populations, and shows that the gene displays varying amounts of positive selection, implying host population genetic differences in the response to *H.pylori *infection, and indicating the benefit of the gene to *H.pylori*. The region of the *cagA *gene under selection contains the EPIYA domains. These observations are an apparent paradox, given the detrimental effects of the oncoprotein on the human host; various scenarios are discussed that may explain the data.

## Methods

### Sequences and phylogenetic analysis

Complete *cagA *sequences from different human populations were obtained from the Genbank database (NCBI) and are listed in Table 1. Although isolated from a white american from Tennessee, the USA sequence has an african origin [29], hence it is denoted African(USA). There were two *cagA *genes in the Peruvian genome, denoted Peru1 and Peru2. There is an additional *cagA *gene in the Venezuelan genome, however this is likely to be a pseudogene because of a 119 amino acid deletion on the N terminus. Searching of the Genbank database, and other *Helicobacter *species did not reveal a significant homolog of cagA. DNA alignments were constructed by first aligning the protein sequences, using the MAFFT program [30], and then using this alignment as a template for a DNA alignment, using the PAL2NL program [31]. Bayesian phylogenetic inference of the *cagA *DNA sequences was conducted using the program MrBayes [32], using a GTR substitution model and a gamma parameter of 0.84, selected using the jModelTest program [33]. The simulation was run for 90000 generations, sampling every 100 generations. A burn-in of 25% was conducted and the consensus tree was constructed from the last 25% of the sampled generations.

**Table 1:** *cagA *sequences used in the study

*H. pylori *strain	Accession number	Origin
26695	GenBank: NC000915	UK

J99	GenBank: NC000921	Africa(USA)

HPAG1	GenBank: NC008086	Sweden

Shi470	Genbank: NC010698; YP001910308 (Peru1), YP001910294 (Peru2)	Peru

G27	GenBank: NC011333	Italy

P12	GenBank: CP001217	Germany

V225	GenBank: CP001582	Venezuela

VietnamHP-No36	GenBank: FJ798973	Vietnam

MEL-HP27	GenBank: DQ306710	Central China

F28	GenBank: AB120418	Japan

3K	GenBank: DQ985738	India

15818	GenBank: AF083352	Austria

42G	GenBank: FJ389581	Hong Kong

Partial rRNA sequences for various *Helicobacter *species were obtained from Genbank; these were *H.fennelliae *(GenBank: AF348747), *H.acinocychis *(GenBank: NR_025940), *H.pylori *(GenBank: DQ202383), *H.nemestrinae *(GenBank: AF363064), *H.heilmannii *(GenBank: AF506794), *H.cetorum *(GenBank: FN565164), *Helicobacter sp. '*solnick 9A1-T71' (GenBank: AF292381), H.biz*zozeronii *(GenBank: NR026372), *H.salomonis *(GenBank: NR026065) and *H.felis *(GenBank: NR025935). The sequences were aligned using the MAFFT program and phylogenetic relationships determined using MrBayes and a HKY model, selected using the jModelTest program. The simulation was run for 10000 generations, sampling every 100 generations. A burn-in of 25% was conducted and the consensus tree was constructed from the last 25% of the sampled generations.

### Positive selection analysis

The *cagA *gene sequences were analyzed for the presence of positive selection by likelihood ratio testing, comparing nested models, null and alternative, using the PAML program [34]. Three tests were performed; the branches test [35,36], sites test [37] and branches-sites test [38]. An unrooted tree without branch lengths was used for the analysis, generated by the phylogenetic analysis, and the codon frequency table option was utilized in all analyses. Likelihood ratio testing was conducted to determine the signficance of 2Δl, the differences between the log likelihoods of the two models (where l is the log likelihood), using a χ^2 ^distribution with 12 degrees of freedom for the branches model, a χ^2 ^distribution and 2 degrees of freedom for the sites model and a χ^2 ^distribution with 1 degree of freedom for the branches-sites model. The null model used for the branches test was a one-ratio model where Ka/Ks (ω) was the same for all branches, while the alternative model was the free-ratio model where ω was allowed to vary. The null model for the sites test was model 1a (neutral; model = 0, NSsites = 1, fix_omega = 0), and the alternative model was model 2a (selection; model = 0, NSsites = 2, fix_omega = 0). The null model for the branches-sites test was modified according to Yang et al. [39] (neutral; model = 2, NSsites = 2, fix_omega = 1, omega = 1). The alternative model was model A (selection; model = 2, NSsites = 2, fix_omega = 0).

## Results and discussion

### Positive selection on cagA

**Figure 1:**
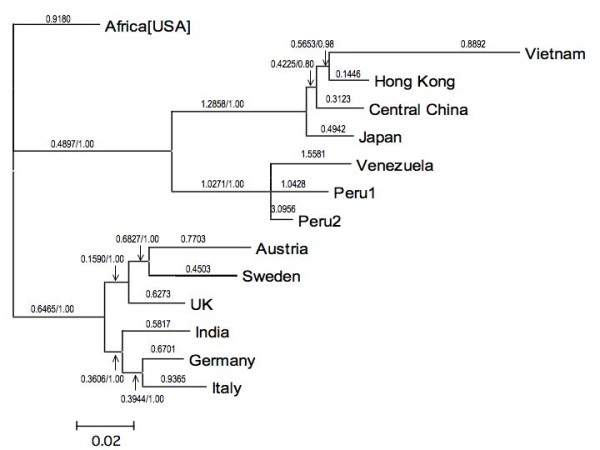
**Positive selection on *cagA *from different *H.pylori *strains**. A phylogenetic consensus tree was constructed as described in Methods using complete *cagA *gene sequences. Numbers above and below branches indicate the values of Ka/Ks calculated for each lineage using the PAML branches test, while numbers after slashes are posterior probabilities of the respective nodes. The scale refers to the average number of substitutions per site.

**Figure 2:**
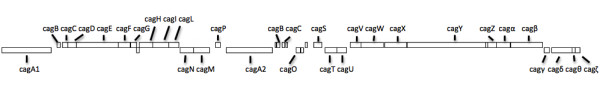
**Diagram of the *cag *PaI from the Peru strain**. Indicated in the figure is the position of the duplicated *cagA *gene.

The topology of the phylogenetic tree of the complete *H.pylori cagA *sequences reproduces the relationships between different human populations around the world (Figure 1), and is consistent with larger scale studies using concatenated sequences that show that *H.pylori *has co-migrated with humans after their exit from Africa [3]. The reproduction of the evolutionary history of the human populations in the topology of the *cagA *tree therefore is the result of the tight association of *H.pylori *with its host [3,40,41]. The *cagA *sequence obtained from an Indian individual is located within the clade formed by european sequences, consistent with results showing that Indian *cagA *sequences intercalate with european sequences [42] and that most *H.pylori *from India are related to european strains [43]. The tree also indicates that the Peruvian *cagA *sequence has undergone a recent gene duplication; this is seen in the operon structure (Figure 2). Strong positive selection on Peru2 indicates that neofunctionalization of the gene is occurring. Presumably, the gene duplication results in gene dosage effects; how this affects the pathogenicity of the strain in unclear. The presence of a pseudogenized *cagA *gene in the *H.pylori *genome isolated from a Venezuelan amerindian (see Methods) is interesting; the reason for the disparity between the fates of the duplicated *cagA *genes in the two related strains is also unclear. The branch lengths on the phylogenetic tree show similarity to each other, with the exception of the Vietnamese lineage; this branch shows considerable accelerated evolution.

2Δl was calculated as 73.6 for the branches test, which was statistically significant. Ka/Ks values of greater than 1 were observed for 5 branches (Figure 1); those leading to the Venezuela (1.56), Peru1 (1.04) and Peru2 (3.10) sequences, to the common ancestor of the amerindian sequences (1.03) and to the lineage leading from the common ancestor of the asian sequences (1.29). These branches are subject to positive selection, while the amerindian common ancestor is neutral over the length of the gene.

2Δl was calculated as 161 between the null and alternative models, for the sites test, which was statistically significant. Estimates of parameters were as follows: p_0 _= 0.51, p_1 _= 0.49, ω_0 _= 0.03, ω_1 _= 1 (neutral model), p_0 _= 0.47, p_1 _= 0.38, p_3 _= 0.14, ω_0 _= 0.03, ω_1 _= 1, ω_2 _= 3.74 (selection model). Sites identified as being under positive selection, with statistical significance according to the Bayes Empirical Bayes test [39], were: 101, 206, 306, 378, 532, 542, 548, 604, 651, 774, 793, 815, 831, 834, 869, 876, 886, 892, 901, 998, 1004. The numbering was based on the Peru1 sequence.

**Figure 3:**
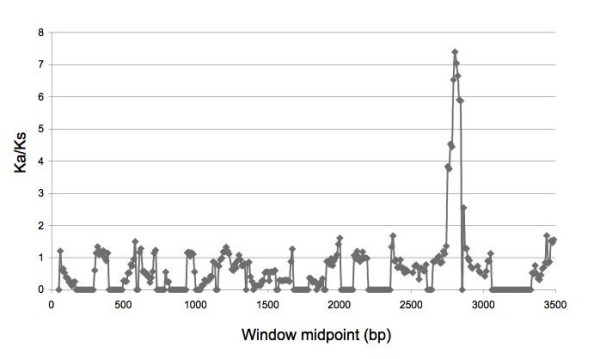
**Sliding window analysis of two *cagA *genes**. Genes from the Venezuela and Peruvian strains (Peru2) were analyzed. Sliding window analysis of a pairwise *cagA *alignment was conducted using the DNASP5.0 program [82], using the Nei and Gojobori [83] method of calculating Ka/Ks. The alignment was constructed as described in Methods. A sliding window of 100 nucleotides, with a step of 10 was used. Gaps were ignored.

A branches-sites test was conducted on each branch of the tree. Those lineages found to display positive selection are listed in Table 2. These included the lineages previously identified by the branches test, and additionally the african, Italian, Swedish and Vietnamese lineages. The results showing positive selection in *cagA *isolated from various populations are consistent with a McDonald-Kreitman test that shows that partial *cagA *sequences isolated from the Mexican population are under positive selection [44]. Parallel evolution in residues or different regions of the cagA proteins is not observed, although residues in the 900 amino acid region are under stronger diversifying selection, when the Venezuelan and Peru2 genes are examined in a sliding window analysis (Figure 3). This is an interesting result as this region of the *cagA *gene encodes the EPIYA repeats, which have a role in modulating the carcinogenicity of the *cagA *gene. Thus, it would appear that the effects of diversifying selection may have a direct role in modulating carcinogenesis.

**Table 2:** Statistics of the branches-sites positive selection analysis

Lineage on tree	2⊗ l	Residues predicted to be under positive selection (p < 0.05)
Venezuela	48.82	794, 834, 837

Vietnam	288.56	202, 274, 275, 277, 278, 279, 281, 282, 283, 287, 461, 834, 895, 896, 899, 900, 901, 903, 905, 908, 910, 911, 912, 913, 914, 915, 916, 917, 918, 919, 920, 921, 922

Sweden	25.6	1008

Peru1	66.2	665, 799, 803

Peru2	3.24	186, 198, 667, 808

Ancestral lineage of Amerindian strains	30	650

Ancestral lineage of Asian strains	21.8	**-**

Africa(USA)	11.42	**-**

Italy	9.52	**-**

Lineages predicted to be under positive selection were identified using the branches-sites test. Residues were identified using the Bayes Empirical Bayes statistic [43], only those that were statistically significant are displayed. Numbering is based on each respective sequence.

### Population specific differences in positive selection

Positive selection on *cagA *is likely to be due to avoidance of the adaptive immune response, IgG, or to enhance binding to cellular receptors which are antagonistically co-evolving. There is a strong immune response against the cagA protein (cagA is immunodominant); this may have led to an 'arms race' between host and bacteria, and hence the signature of positive selection. This is often the case with extracellular proteins of pathogens, either located on the cell surface or secreted. There is a precedent in bacteria, with the *porB *porin gene of *Neisseria gonorrhoeae *and *meningitidis *[45], and a variety of extracellular proteins from *Escherichia coli *[46]. Secreted slr proteins from *H.pylori *also show signatures of positive selection [47]. This scenario would imply that the regions of *cagA *under positive selection are immunogenic.

*H.pylori cagA *from a range of populations around the world show evidence of positive selection (using the branches-sites test); these include sequences from Venezuela, Vietnam, Sweden, Peru, Africa and Italy. However, as human and *H.pylori *strains have co-evolved, *cagA *genes from some strains have undergone stronger positive selection, particularly the strains with ancestry in the human groups that most recently migrated, the asians and the amerindians [48,49]. The cause of the differences in strength of selection on the *cagA *genes presumably lies in genetic differences at the host level, but is also potentially mediated by different responses induced by the cagA protein, resulting from functional differences between different cagA proteins. The intra-population genetic distances are smaller in human groups as they migrated east out from Africa [50]. Host-specific differences may include differences in the immune response, or differences in the activities of cellular cagA binding proteins. Codon usage analysis (Table 3) indicates that the codon adaptation index is similar for different *cagA *genes, suggesting that there are no strong differences in translational selection between *cagA *genes from different *H.pylori *strains, which may indicate no major functional differences between genes or simply reflect the lack of translational selection on highly expressed genes genome-wide [51]. This data helps to inform the sliding window analysis; translational selection has been shown to result in false indications of positive selection [52]: this is not likely to be the case here due to the lack of translational selection on these genes.

**Table 3:** Codon usage analysis of the *cagA *genes

Gene	*H. pylori *Strain	CAI
UK	26695	0.699

Africa (USA)	J99	0.697

Sweden	HPAG1	0.695

Peru1	Shi470	0.712

Peru2	Shi470	0.698

Italy	G27	0.693

Germany	P12	0.690

Venezuela	V225	0.702

Vietnam	VietnamHP-No36	0.695

Central China	MEL-HP27	0.700

Japan	F28	0.700

India	3K	0.691

Austria	15818	0.686

Hong Kong	42G	0.701

The codon adaptation index (CAI) was calculated as follows. A codon usage table for the complete *H.pylori *genome of strain HPAG-1, which comprised 1544 ORFs, was obtained from the Codon Usage Database http://www.kazusa.or.jp/codon. This was used to calculate the CAI for each individual ORF.

Polymorphisms in the IL-1 gene cluster modify gastric cancer risk [53]. The induction of IL-8 secretion by the *cag *PaI is a major stimulus of the immune response [49]. Thus, differences in host interleukin genotypes may lead to differences in outcome for disease progression and differences in selective pressure on the *cagA *genes in different populations. Amerindians underwent a population bottleneck during the migration of their ancestors from Asia [48]. Phenotypic evidence of this is the universality of the O blood group amongst amerindians [54], this may have led to a homogeneity of immune response. This may have affected the strains capacity to bind non O human blood antigens; most *H.pylori *strains are able to bind the A,B and O antigens via the babA adhesin, while amerindian strains from South America bind best to O antigens [55]. It is interesting to note that the east asian population is also relatively genetically homogenous [49].

Both commensal and pathogenic bacteria possess mechanisms for the avoidance of the host immune system. Several mechanisms have been shown to be involved in avoidance of the immune system by *H.pylori*. However, *cagA*+ strains elicit a strengthened immune response and increased inflammation [56-58]. Inflammation may be a mechanism to obtain nutrients [59], however if *cagA *is evolving to avoid the immune system while at the same time stimulating it, then this seems contradictory.

### Distribution of gastric cancer worldwide and its relationship with the strength of positive selection on cagA

There are great variations in the incidence of gastric cancer worldwide, with parts of East Asia and Latin America showing high incidences, while other parts of the world such as Africa and parts of Europe showing low incidences (Table 4). The incidence rates do not correlate with rates of infection with *H.pylori*. For instance, there are high rates of *H.pylori *associated pathogenicity in Japan, Korea and parts of China, but low in Thailand and Indonesia even though they have high infection rates; this is the 'Asian paradox' [60]. Instead, incidence appears to be linked to the frequency and genotype of *cagA *[61], while other factors are also likely to play a role such as altitude, diet and host genotype. In addition, recent work shows that recent migrations and population movements have resulted in the introduction of 'non-native' *H.pylori *strains with different *cagA *alleles into established human populations [42,62], this gives an added level of complexity.

**Table 4:** Mortality figures from gastric cancer for populations examined in this study

Region	Incidence of gastric cancer (per 100000)	Incidence of esophageal cancer (per 100000)
Peru	21.2	1.1

Venezuela	10.4	1.5

Japan	31.1	5.7

Central China	29.9	16.7

Hong Kong	29.5	12.7

Vietnam	18.9	1.9

Austria	7	2.6

Germany	7.7	3.8

India	3.8	5.3

Italy	10.9	1.9

Sweden	4.3	2.2

U.K.	5.6	6.6

Africa	4	5

Data were derived from the GLOBOCON project, International Agency for Research on Cancer, World Health Organization and Center for Health Protection, Department of Health, Government of the Hong Kong Special Administrative Region (Hong Kong).

Given that amerindian and the ancestral asian *cagA *sequences show stronger signs of positive selection, and that asian and latin american populations can exhibit high incidences of gastric cancer, this might imply a link between the strength of positive selection on the *cagA *gene and the oncogenicity of the gene. The results of the sliding window analysis, where the cagA region containing the EPIYA domains is under positive selection, are consistent with this hypothesis. Further work is required. If verified, this form of sequence analysis may help identify at risk populations.

### Evolutionary benefit of cagA to *H.pylori*

The signature of positive selection observed on the *cagA *gene indicates that the cagA protein is undergoing adaptive evolution in some strains, and is beneficial to the bacteria. Differences in rates of adaptation imply host specific differences. The benefit to the bacteria is mediated via the role of *cagA *within the pathogenicity island; the specific role of *cagA*, and that of the PaI, remain to be determined. In general, PaIs have a role in promoting survival of bacterial pathogens [63]. The positive selection observed on the *cagA *oncogene is unusual as it is the first case observed of positive selection on an oncogene in a vertically transmitted pathogen. Positive selection is a feature of antagonistic coevolution, which implies harmful effects on the host, but also mutualistic coevolution, which implies benefits. Positive selection has been observed on the Epstein Barr Virus - encoded oncogene LMP1 [64] and the human papillomavirus type 16 oncogene [65,66], however these are horizontally transmitted pathogens where a balance is expected between virulence and transmissibility [67]. This may imply that *H.pylori *has been horizontally transmitted to a greater extent than previously recognized.

**Figure 4:**
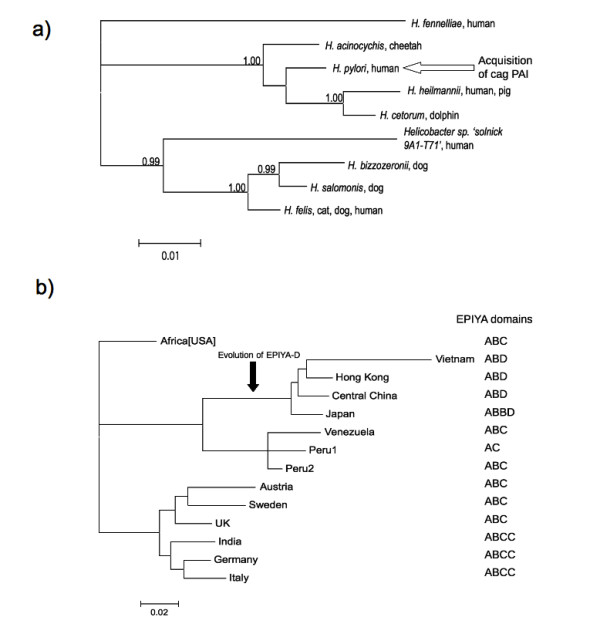
**Genetic factors leading to an increase in virulence of *Helicobacter pylori***. a) Small subunit rRNA phylogenetic consensus tree of bacterial species in the human digestive system related to *H.pylori*, showing the recent acquisition of *cagA*; b), the EPIYA domains that are present in the *cagA *gene and the evolution of the EPIYA-D domains in the asian lineages. Tree (a) was constructed as described in Methods, numerals indicate posterior probabilities, tree (b) as in Figure 1.

Virulence is a result of enhanced reproduction of a pathogen. Early models proposed that a parasite would be inclined to evolve reduced virulence, given that mortality of host is a disadvantage. However, this view has been criticized as relying on group selection [68]. However, vertically inherited pathogens are expected to become less pathogenic over time; if the pathogen depends on the host for transmission and the transmission is highly efficient then it is not in the interests of the pathogen to significantly reduce the fitness of the host [69]. *H.pylori *displays two features, in addition to the positive selection observed on *cagA*, that appear to contradict this paradigm. Firstly, the acquisition of the *cag *PaI during speciation from related non-pathogenic gut helicobacters (Figure 4a), indicates that *H.pylori *underwent an initial increase in pathogenicity. Second, the evolution of the more pathogenic EPIYA-D motifs in the *cagA *gene in some asian strains (Figure 4b), indicates that some *cagA+ H.pylori *has undergone a more recent additional increase in pathogenicity. To some extent, this contradiction could be explained by the proposal that there is actually a host - beneficial component to cagA, or that it has not exerted a sufficiently deleterious effect on the host. One question that requires answering is whether those strains that are undergoing a greater degree of positive selection are becoming more pathogenic.

In addition, potential beneficial effects of *cagA *at the population level via elimination of the elderly has been suggested [13] (this explanation relies on the theory of inclusive fitness [70]). This essentially views *cagA *as a gene that enhances intrinsic mortality in old individuals, however it is unclear whether intrinsic mortality in a subgroup of the population has ever been selected for. While *H.pylori *has largely been considered a pathogen, there is increasing evidence of its positive benefits to human health. For instance, *H.pylori *has a beneficial role in preventing esophageal cancer, by reducing acid reflux [71,72], however in the past this has been unlikely to have provided much evolutionary benefit to the human population given that over 90% of patients are over 55 [73], while before the 20^th ^century the average life expectancy of human populations was less than 40. The strongest inverse correlation between esophageal cancer occurrence and infection with *H.pylori *is in East Asia, attributed to the highly interactive (eastern) form of *cagA*, which causes pan- and corpus- predominant gastritis and reduces acid production [13]. There is also an inverse relationship between *H.pylori *and asthma and allergies [74-76], obesity [77] and infant diarrhea [78]. Asthma and obesity are modern illnesses, so are unlikely to have played a role in the evolutionary dynamics of the bacteria.

Ulcers are a modern disease [79], while gastric cancer has been recorded since ancient times. However, it is most prevalent in 55 year olds and over, this indicates that historically it is unlikely to have exerted a strong selective pressure, given that before the 20^th ^century the average life expectancy was considerably lower. These considerations lead to the conclusion that the *cagA *gene is either insufficiently deleterious to the human host, that the cagA protein has a beneficial component to the host, or that horizontal transmission has been an important feature of *H.pylori *in the recent past. There is increasing evidence that in developing countries, horizontal transmission of *H.pylori *occurs due to poor sanitary conditions [80,81]. If there is (or has been) significant horizontal transmission, then there may be population specific differences in the amount of horizontal transmission which may have led to differences in selective pressures on the pathogen.

*H.pylori *has been utilized as a model for infective carcinogenesis, and is a model of pathogen evolution. The results of this work suggest that the *cagA *gene is insufficiently deleterious to the human host, that the cagA protein has a benefit to the host or that horizontal inheritance has affected the evolutionary dynamics of the bacteria more than recognized. The results reported here offer an insight into important aspects of microbe-host coevolution.

## Competing interests

The authors declare that they have no competing interests.

## Authors' contributions

GD and SM conducted the analyses, MD and SM designed the study. All authors read and approved the final manuscript.
